# Impaired orthostatic blood pressure recovery and cognitive performance at two-year follow up in older adults: The Irish Longitudinal Study on Ageing

**DOI:** 10.1007/s10286-016-0340-3

**Published:** 2016-03-11

**Authors:** Joanne Feeney, Neil O’Leary, Rose Anne Kenny

**Affiliations:** Centre for Public Health, Institute of Clinical Sciences, Block B, Queens University Belfast, Royal Victoria Hospital, Grosvenor Road, Belfast, BT12 6BA UK; The Irish Longitudinal Study on Ageing, Trinity College Dublin, Dublin 2, Ireland

**Keywords:** Orthostatic hypotension, Cognition, Ageing, Population, Longitudinal

## Abstract

**Background:**

Prospective investigations of the association between impaired orthostatic blood pressure (BP) regulation and cognitive decline in older adults are limited, and findings to-date have been mixed. The aim of this study was to determine whether impaired recovery of orthostatic BP was associated with change in cognitive function over a 2-year period, in a population based sample of community dwelling older adults.

**Methods:**

Data from the first two waves of the Irish Longitudinal Study on Ageing were analysed. Orthostatic BP was measured during a lying to standing orthostatic stress protocol at wave 1 using beat-to-beat digital plethysmography, and impaired recovery of BP at 40 s post stand was investigated. Cognitive function was assessed at wave 1 and wave 2 (2 years later) using the Mini-Mental State Exam (MMSE), verbal fluency and word recall tasks.

**Results:**

After adjustment for measured, potential confounders, and multiple imputation for missing data, the change in the number of errors between waves on the MMSE was 10 % higher [IRR (95 % CI) = 1.10 (0.96, 1.26)] in those with impaired recovery at 40 s. However, this was not statistically significant (*p* = 0.17). Impaired BP recovery was not associated with change in performance on any of the other cognitive measures.

**Conclusions:**

There was no clear evidence for an association between impaired recovery of orthostatic BP and change in cognition over a 2-year period in this nationally representative cohort of older adults. Longer follow-up and more detailed cognitive testing would be advantageous to further investigate the relationship between orthostatic BP and cognitive decline.

**Electronic supplementary material:**

The online version of this article (doi:10.1007/s10286-016-0340-3) contains supplementary material, which is available to authorized users.

## Introduction

Orthostatic hypotension (OH) is the term given to an exaggerated drop in blood pressure (BP) on standing from a seated or lying (supine) position. The classical definition of OH is a decrease in at least 20 mmHg systolic and/or 10 mmHg diastolic BP within 3 min of standing [1], usually assessed by oscillometric measurement. OH is more common among older adults and is associated with an increased risk of all-cause mortality [2]. Recently, there has been heightened interest not only in the extent of the drop in BP on stand, but also on the time taken for BP to return to baseline (pre-stand) levels. Usually BP returns to baseline within around 30 s after standing [3]. Continuous, beat-to-beat monitoring of BP allows detailed investigation of patterns of recovery. Individuals exhibiting delayed recovery after standing are more likely to be frail [4] and a failure to recover BP to baseline within a minute after standing has been associated with incident mortality in the subsequent 5 years among older adult falls clinic attendees [5].

Although the mechanisms governing cerebral perfusion are not fully understood, there is evidence that blood flow to the brain can be compromised during an episode of OH [6]. Furthermore, older adults may be at greater risk of hypoperfusion during hypotensive episodes than younger adults due to impaired cerebral autoregulation [6, 7]. Decreases in cerebral blood flow velocity have been associated with dementia [8] and amnestic cognitive impairment [9]. Moreover, impaired orthostatic blood pressure (OBP) recovery in individuals with mild cognitive impairment has been shown to predict conversion to dementia [10]. Transient decreases in cerebral perfusion due to impaired OBP regulation may deprive neuronal cells of vital oxygen and glucose, causing cell damage and leading to cognitive decline [11]. Importantly, impaired OBP regulation is treatable and represents a modifiable risk factor for cognitive impairment. Early intervention may be crucial to reduce the risk of cognitive decline. Therefore investigations of the association between OBP regulation and cognition in community dwelling middle-aged and older adult cohorts are warranted.

A limited number of studies have examined the relationship between impaired orthostatic BP regulation and cognitive function in non-clinical cohorts of older adults and findings have been somewhat contradictory [12–14], possibly due in part to differences in sample sizes and characteristics, BP and cognitive measures. Using continuous BP measurement previous research from our group has shown a cross-sectional association between impaired recovery and cognitive function among individuals with supine hypertension [15]. Prospective investigations of the association between OBP behaviour and cognitive function in large samples using this sensitive measure of BP assessment are lacking. The aim of the current study was thus to determine whether impaired recovery of OBP, as assessed by beat-to-beat measurement was associated with a decline in cognitive function over a two-year period, in a population based sample of community dwelling older adults.

## Methods

Data from the first two waves of The Irish Longitudinal Study on Ageing (TILDA) were analysed. TILDA is a longitudinal cohort study of adults aged 50 and older in Ireland who were living in the community at the study outset. Wave 1 took place between 2009–2011 and wave 2 was carried out in 2012–2013. 8175 individuals aged 50 and older took part in wave 1 and 7282 participated in wave 2. Detail of the study design is published elsewhere [16]. Briefly, the data collected comprised: (1) a computer assisted personal interview (CAPI) carried out in the participant’s own home; (2) a self-completion questionnaire; and (3) a health assessment carried out by trained nurses in a dedicated centre. At wave 2, for any individuals who were no longer cognitively or physically capable of being interviewed in person, a proxy interview was carried out whereby a nominated spouse, family member or carer was interviewed on behalf of the individual. 80 proxy interviews were conducted at wave 2.

### Blood pressure

OBP was measured during the health assessment at wave 1 using beat-to-beat digital plethysmography (Finometer MIDI; Finapres Medical Systems, Arnhem). Subjects underwent a lying-to-standing orthostatic stress test. For this test, participants lay in a supine position for at least 10 min before stand. Beat-to-beat recordings of 180 s in duration, beginning 60 s before stand and lasting 120 s after stand, were taken. Orthostatic BP processing required a number of steps including: (a) data quality screening and artefact rejection; (b) preprocessing and filtering; and (c) BP waveform feature extraction as described previously [4]. BP was estimated in 10 s intervals using 5 s moving averages around each point to smooth the beat-to-beat BP variations. Recently, impaired OBP recovery at 40 s post stand has been shown to be a particularly sensitive indicator of impaired BP stabilisation after standing and is more common among older age groups [17]. We thus calculated the extent of the drop in systolic and diastolic BP from baseline (supine, pre-stand) level at 40 s post stand and characterised any individuals with a drop of ≥20 mm Hg systolic and/or 10 mmHg diastolic BP as exhibiting impaired recovery.

### Cognitive function

Cognition was assessed at wave 1 and at wave 2, 2 years later. The measures included in both waves were the Mini-Mental State Exam (MMSE), verbal fluency, and a 10-word recall task. The MMSE [18] is a global test of cognitive function in widespread use in both clinical and non-clinical settings. It tests several domains of cognition and the maximum score is 30. The verbal fluency task administered at both waves was a standard category fluency task which required participants to name as many animals as possible in 1 min. The score is the total number of animals named within this timeframe. This task assesses executive functions such as planning and mental flexibility, and also relies on intact semantic memory [19]. Immediate and delayed memory were assessed using a ten-word list recall previously developed for the Health and Retirement Survey [20]. Participants were asked to listen to 10 words and then immediately recall them. This sequence was repeated again and the score for both recall tests was summed to give a total immediate recall score. After a short delay individuals were asked to recall the ten words again, this time without hearing them immediately prior (delayed recall).

### Covariates

Covariates selected from those available were putative risk factors for cognitive decline, or variables previously associated with cognitive performance, but did not have a rate of missingness of greater than 10 % in the sample of interest. Information on age, sex, educational attainment (primary/none, secondary, or third level), doctor diagnosed diseases, health behaviours and medication usage was gathered at both wave 1 and wave 2. Individuals were asked about their yearly household income categorised into five bands (Less than €10,000, between €10,000 and €20,000, between €20,000 and €40,000, between €40,000 and €70,000, and greater than €70,000). Symptoms of depression and problem drinking were assessed using the centre for epidemiological studies depression scale (CES-D) [21] and the CAGE scale [22], respectively. At both wave 1 and wave 2 all participants were asked whether they had received a doctor’s diagnosis of any of the following conditions: heart attack, angina, heart failure, stroke, transient ischaemic attack, arrhythmia, diabetes, heart murmur. Self-reported medication use was recorded during the CAPI and confirmed by cross-checking with medication labels. Medications were classified according to the Anatomical Therapeutic Classification (ATC) (http://www.whocc.no/atc_ddd_index/). A dichotomous variable was generated to indicate usage of any cardiovascular medication, defined as any of the following classes of drugs: cardiac therapy (‘C01’), antihypertensives (‘C02’), diuretics (‘C03’), peripheral vasodilators (‘C04’), vasoprotectives (‘C05’), beta blockers (‘C07’), calcium channel blockers (‘C08’), ACE inhibitors (‘C09’) and lipid modifying agents (‘C10’). Psychoactive medications were also classified. They were defined as nervous system drugs comprising anti-Parkinson drugs (‘N04’), psycholeptics (‘N05’) and psychoanaleptics (‘N06’) and parasympathomimetics (‘N07A’).

### Exclusion criteria

Any individuals who self-reported a doctor’s diagnosis of stroke, Parkinson’s disease or dementia at wave 1 were excluded from the analysis. A significant proportion (*n* = 3039) of the wave 1 sample did not undergo a health assessment and thus were necessarily excluded. Furthermore, 56 individuals died between waves and therefore were not in the target sample for analysis of change.

**Fig. 1:**
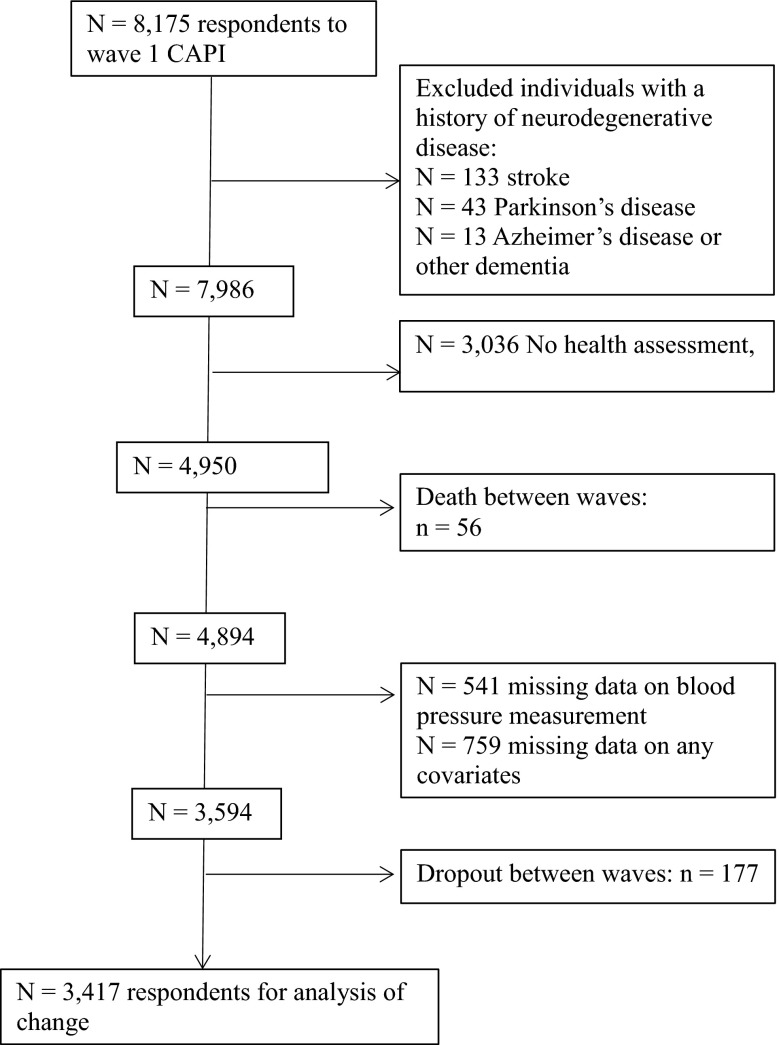
Sample for analyses

Figure 1 details the number of individuals excluded, the number with missing data and the final observed sample for analysis. Missing data was imputed using chained multiple imputation (for full description of the procedure see Online Supplementary Material).

### Data analysis

The distribution of all variables of interest was initially examined via histograms and Q–Q plots. Bivariate tests of association between OH and the other variables of interest were carried out using *t* tests, Mann–Whitney *U* and Chi-squared tests as appropriate. Analysis of the association between baseline OBP behaviour, baseline cognition and change in cognition at wave 2 was carried out using mixed-effects regression (ME) modelling. ME models offer some advantages over more traditional techniques like repeated-measures ANOVA in that they allow an imbalance between individuals in the number of repeat measurements and continuous covariates to be included. A single ME model was fitted for each outcome of interest: MMSE errors, verbal fluency score, immediate recall and delayed recall scores. In the case of the MMSE, on account of ceiling effects on the task, the number of errors was calculated (30—total score achieved). This count variable was then modelled using negative binomial ME regression. Linear ME models were applied for the other cognitive outcomes. The variables entered as predictors in all ME models were impaired OBP recovery 40 s (measured at wave 1) as the predictor of interest, an indicator variable for wave (1 or 2), and other covariates at wave 1 as defined previously: age, sex, education, income, medication usage, cardiovascular conditions, CES-D score, and problem drinking. Age was included as a continuous covariate in all models as a restricted cubic linear spline to account for a curvilinear relationship of cognition with age. In addition to the impact of these variables on cognition at baseline, terms modelling the effect of all baseline variables on change in cognition over time were also included by entering an interaction term for each of the other variables with wave.

Analyses were carried out using Stata 12.0 and statistical significance was set at *p* < 0.05, but Holm–Bonferroni corrected where variables of interest were derived from the same domain (i.e., immediate and delayed word recall scores).

## Results

### Demographic characteristics of the observed sample

Investigation of the individuals with complete data observed differed from those who were missing data with respect age and education. Observed cases were younger [mean (SD) = 61.6 (8.2) versus 65.1 (10.2), *p* < 0.001], and were more likely to have stayed longer in full time education than cases with missing data (38.6 % had attained tertiary level education or higher versus 22.9 % among cases with missing data, *p* < 0.001). Individuals with full data observed were also slightly more likely to be male (46.9 % versus 44.6 %, *p* = 0.04).

### Wave 1 characteristics of the sample by impaired orthostatic BP recovery

Table 1 shows the wave 1 characteristics of the sample by impaired OBP recovery at 40 s post stand. Individuals exhibiting impaired recovery at this time point were older (*p* < 0.001), more likely to be female (*p* = 0.03), taking cardiovascular (*p* = 0.003) and psychotropic medications (*p* < 0.001). They were also likely to have a lower yearly household income than those with normal recovery (*p* < 0.001). No significant difference between the groups was observed in the prevalence of problem drinking or depressive symptoms in the descriptive analyses.

**Table 1:** Wave 1 demographic and health covariates by impaired orthostatic BP recovery at 40 s

Variable	No impaired OBP *n* = 2970	Impaired OBP *n* = 446	*p*
Mean (SD)/% (*n*)			
Age	60.9 (7.9)	65.4 (9.1)	<0.001
Female	52.4 (1557)	57.8 (258)	0.03
Education			
Primary/none	18.6 (552)	28.0 (116)	
Secondary	42.7 (1267)	36.1 (161)	
Tertiary	38.7 (1151)	37.9 (169)	0.001
Problem drinking	14.7 (437)	13.0 (58)	0.34
Missing % (*n*)	9.1 (345)	10.0 (60)	
Cardiovascular medication use	45.4 (1348)	52.9 (236)	0.003
Psychotropic medication use	7.3 (218)	13.9 (62)	<0.001
History of cardiovascular condition	11.4 (338)	14.1 (63)	0.09
CES-D score	5.2 (6.3)	5.4 (6.9)	0.47
Missing % (*n*)	1.2 (44)	1.2 (7)	
Yearly household income (euro)			
Less than 10,000	7.4 (230)	8.7 (39)	
Between 10,000 and 20,000	14.2 (422)	19.1 (85)	
Between 20,000 and 40,000	35.6 (1058)	41.7 (186)	
Between 40,000 and 70,000	28.5 (846)	19.7 (88)	
Greater than 70,000	13.9 (414)	10.8 (48)	<0.001
Missing % (*n*)	7.6 (291)	8.5 (51)	

### Cognitive scores at wave 1 and wave 2

There was no evidence of any meaningful difference in unadjusted MMSE scores for the sample between wave 1 and wave 2 [wave 1 median (IQR) = 29 (28–30); wave 2 median (IQR) = 29 (28–30)]. There were statistically significant differences in immediate word recall and verbal fluency scores between waves, with the former showing a slight improvement over time and the latter, a slight decline [immediate recall, wave 1 mean (SD) = 13.4 (3.2), range 2–20; wave 2 mean (SD) = 13.6 (3.2), range 0–20, *p* < 0.001; verbal fluency, wave 1 mean (SD) = 20.8 (7.0), range 0–50, wave 2 = 19.2 (6.1), range 0–45, *p* < 0.001]. Performance on the delayed word recall task did not differ significantly between waves [wave 1 mean (SD) = 6.0 (2.3), range 1–10; wave 2 mean (SD) = 6.0 (2.6), range 1–10, *p* = 0.14].

### Comparison of individuals with data fully observed versus those with imputed data on variables with missingness

Comparison of those individuals for who missing data was imputed and individuals with data fully observed on the exposure, covariates and outcomes of interest revealed some differences (Supplementary Data). Individuals in the fully observed sample were less likely to have OH at 40 s post stand. They also had lower scores on the CES-D scale and were less likely to be classified as problem drinker according to the CAGE scale than individuals for whom data was missing and subsequently imputed. However, individuals with data fully observed had higher scores on each of the cognitive tests at wave 2 and had a wider range of income than the imputed cases.

### Multivariable analyses of the association between wave 1 orthostatic blood pressure recovery and change in cognitive scores between waves

The association between baseline OBP recovery and cognitive performance from ME regression models using the full sample of interest (both observed and imputed data) is displayed in Table 2. In the case of verbal fluency, immediate and delayed recall scores, linear coefficients are displayed and correspond to the change in cognitive score associated with a unit change in the predictor. In each case a negative coefficient reflects a decrease in cognitive scores. As MMSE was analysed in terms of the number of errors, the coefficient is an incident rate ratio (IRR), or the change in rate ratio of MMSE errors associated with a unit increase in the predictor. Thus a positive coefficient in this case reflects a decrease in performance. Impaired OBP recovery at 40 s at wave 1 was not associated with cognitive performance at wave 1 or wave 2 after adjustment for both cross-sectional and longitudinal associations between the other covariates and cognition. Although the change in the number of errors between waves on the MMSE was 10 % higher in individuals with impaired recovery at 40 s [IRR (95 % CI) = 1.10 (0.96, 1.26)], this was not statistically significant (*p* = 0.17) (Table 2). Based on adjusted mean marginal estimates, MMSE performance for the total sample improved between wave 1 and wave 2 (change in number of errors: –0.20, 95 % CI –0.24, –0.15, *p* < 0.001). Adjusted mean marginal performance on the immediate recall task also improved by 0.31 points (95 % C.I. 0.20, 0.42, *p* < 0.001) between waves. However, verbal fluency declined by 1.78 points (95 % CI –2.03, –1.54, *p* < 0.001). The mean marginal change in delayed recall score between waves was not significant (0.2 points, 95 % CI –0.06, 0.11, *p* = 0.63).

**Table 2:** Results of the mixed-effects models on imputed data showing the effect of wave and orthostatic BP recovery on cognitive function

		MMSE errors		Verbal fluency		Immediate recall		Delayed recall
	IRR (95 % CI)	*p*	B (95 % CI)	*p*	B (95 % CI)	*p* ^**#**^	B (95 % CI)	*p* ^**#**^
Impaired recovery (40 s)	1.01 (0.90,1.13)	0.91	–0.25 (–0.78,0.29)	0.36	–0.07 (–0.31,0.17)	0.72	–0.09 (–0.29,0.11)	0.72
Wave (2 versus 1)	0.80 (0.67,0.97)	0.02	–3.36 (–4.17, 2.56)	<0.001	0.81 (0.43,1.19)	<0.001	–0.05 (–0.34,0.24)	0.72
Impaired recovery X wave	1.10 (0.96,1.26)	0.17	–0.19 (–0.36,0.75)	0.49	0.10 (–0.16,0.37)	0.45	0.14 (–0.06,0.35)	0.34

^#^ *P*-values were Holm–Bonferroni corrected for similar hypotheses for each effect of interest

### Sensitivity analysis

To test the robustness of our findings to the definition and timepoint used, we took a measure of more markedly impaired recovery as our predictor of interest, namely the failure to recover at least 80 % of baseline (supine, pre-stand) BP by 60 s post stand. This categorisation has previously been shown to predict incident mortality among falls clinic outpatients [5]. There was some evidence that individuals who had failed to recover at least 80 % of their baseline systolic BP by 60 s post stand at wave 1 made more errors on the MMSE at wave 2, but again this association was not statistically significant [IRR (95 % CI) 1.22 (0.97, 1.54), *p* = 0.09]. None of the other cognitive variables showed any relationship with this definition of impaired recovery.

## Discussion

After adjustment for wave 1 covariates and their relationship with change in cognition over time, there was no clear evidence for an independent relationship between impaired orthostatic BP recovery in the first-min after standing and cognitive performance at follow-up assessment 2 years later, in a large population representative group of community dwelling older adults. To our knowledge this is the first study to investigate the relationship between the orthostatic BP response and change in cognition over time in a large nationally representative sample using beat-to-beat BP measurement.

There is evidence linking low BP generally in late life to cognitive decline and dementia [23, 24] as well as to the hypoperfusion of subcortical regions and frontal regions of the brain [25, 26]. The extent to which repeated transient drops in BP, such as those observed in orthostatic hypotension, cause hypoperfusion in older adults is an important topic for further investigation as it may have negative consequences for brain health. From a mechanistic perspective, prolonged drops in BP on standing leading to reduced blood flow in older adults likely increases the risk of cognitive impairment by causing or exacerbating existing ischaemic white matter lesions [27]. This risk may be higher among those with low cognitive reserve or pre-existing pathology. Furthermore, age-related arterial stiffening and atherosclerotic plaque deposition may worsen hypoperfusion while simultaneously increasing the risk of further orthostatic hypotension via impairment of baroreflex function [28]. An alternative hypothesis holds that early neurodegenerative processes may result in dysregulation of the autonomic nervous system [29, 30] which may in turn impact negatively on both BP regulation and cognitive decline, thereby explaining the previously observed association between these two phenomena. As the cohort of older adults in the current analysis excluded anyone with overt dementia or cognitive impairment, and average cognitive scores were high, our finding of no prospective association between orthostatic BP recovery and cognition may lend support to this latter hypothesis.

Several important caveats should be noted when interpreting the absence of a longitudinal relationship between OBP recovery and cognitive function. First, the extent of any change in cognition over 2 years could be expected to be minimal especially given that this is a relatively healthy sample (no overt dementia at baseline), and learning effects similar to those observed on the MMSE and immediate word recall tasks have previously been demonstrated in ageing cohorts over short to medium time frames [31]. A longer re-test period and a more comprehensive suite of cognitive tests would be advantageous to examine the relationship between OBP behaviour and cognitive decline more thoroughly. Second, some degree of measurement error is always inherent in repeated physiological or functional assessments. When a limited number of measurements are available to investigate change over time, measurement error can attenuate any underlying associations [32]. Related to this is the potential for an effect of regression to the mean on our longitudinal estimates. Though this should be acknowledged, we believe that impaired OBP regulation is likely to be more closely associated with the long-term change in cognition rather than cognitive performance as assessed contemporaneously, and therefore regression to the mean effects are less of a concern.

Strengths of the current study include the use of finometry in the assessment of blood pressure behaviour, the large sample of older adults assessed, the wide range of potential confounding variables accounted for, and the statistical techniques employed to minimise bias in terms of selection into the sample for longitudinal analysis. Full health assessments in TILDA are conducted only every other wave, so we did not have the full suite of cognitive tests at our disposal for analysing 2-year change. However, the cohort will be tested again at 4 years from wave 1 with a more comprehensive suite of cognitive tests along with another measurement of OBP and we will attempt to explore the relationship between these variables in greater depth.

## Electronic supplementary material

Below is the link to the electronic supplementary material.

Supplementary material 1 (DOCX 18 kb)
