# Supplementary material for: Impaired orthostatic blood pressure recovery and cognitive performance at two-year follow up in older adults: The Irish Longitudinal Study on Ageing
Source: Clin Auton Res. 2016 Mar 11;26:127–33. doi: 10.1007/s10286-016-0340-3 (PMC4819922; doi:10.1007/s10286-016-0340-3)
Supplement: Supplementary file 1 — Supplementary material 1 (DOCX 18 kb) [file 10286_2016_340_MOESM1_ESM.docx]

Missing data and multiple imputation

Missing data was imputed using chained multiple imputation. For participants who attended the health centre assessment and who were within selection criteria, multiple imputation by chained equations^28,29^ was employed to impute partially missing covariates including the determination of impaired OBP recovery and the cognitive outcomes for those participants not known to have died but lost to follow-up between wave 1 and wave 2. All available covariate and outcome values included the substantive analysis model were used to impute other missing values either through predictive mean matching or regression models with truncation where appropriate. Blood pressure variables from the active stand were imputed as continuous variables before being recoded as categorical OH variables. Other covariates included in the imputation model, but not in the substantive analysis were geographic location, household size, social class, employment, marital status, self-rated physical health and self-rated mental and emotional health, and smoking history. Our findings would thus be robust to any missingness which could be explained by this large set of variables.

**Table S1** **A descriptive comparison of individuals with data fully observed versus those with data imputed on variables with missingness.**

| **Variable** | **Observed cases** | **Imputed cases** |
| --- | --- | --- |
|  | **% / Mean** | |
|  |  |  |
| Impaired OBP recovery at 40 seconds | 13.6 | 18.6 |
| Wave 2 cognitive scores |  |  |
| MMSE errors | 1.1 | 1.6 |
| Verbal fluency | 19.9 | 18.7 |
| Immediate recall | 14.2 | 13.5 |
| Delayed recall | 6.3 | 5.8 |
| Covariates |  |  |
| CES-D score (mean) | 5.5 | 8.8 |
| Problem drinking | 13.8 | 17.9 |
| Yearly household income (euro)  Less than 10,000  Between 10,000 and 20,000  Between 20,000 and 40,000  Between 40,000 and 70,000  Greater than 70,000 | 8.3  16.1  36.0  26.8  12.7 | 0.5  6.2  57.0  33.6  2.6 |
